# Nation-Wide, Web-Based, Geographic Information System for the Integrated Surveillance and Control of Dengue Fever in Mexico

**DOI:** 10.1371/journal.pone.0070231

**Published:** 2013-08-06

**Authors:** Juan Eugenio Hernández-Ávila, Mario-Henry Rodríguez, René Santos-Luna, Veronica Sánchez-Castañeda, Susana Román-Pérez, Víctor Hugo Ríos-Salgado, Jesús Alberto Salas-Sarmiento

**Affiliations:** 1 National Institute of Public Health of Mexico, Cuernavaca, Morelos, Mexico; 2 Public Health School of Mexico, Cuernavaca, Morelos, Mexico; University of Malaya, Malaysia

## Abstract

Dengue fever incidence and its geographical distribution are increasing throughout the world. Quality and timely information is essential for its prevention and control. A web based, geographically enabled, dengue integral surveillance system (Dengue-GIS) was developed for the nation-wide collection, integration, analysis and reporting of geo-referenced epidemiologic, entomologic, and control interventions data. Consensus in the design and practical operation of the system was a key factor for its acceptance. Working with information systems already implemented as a starting point facilitated its acceptance by officials and operative personnel. Dengue-GIS provides the geographical detail needed to plan, asses and evaluate the impact of control activities. The system is beginning to be adopted as a knowledge base by vector control programs. It is used to generate evidence on impact and cost-effectiveness of control activities, promoting the use of information for decision making at all levels of the vector control program. Dengue-GIS has also been used as a hypothesis generator for the academic community. This GIS-based model system for dengue surveillance and the experience gathered during its development and implementation could be useful in other dengue endemic countries and extended to other infectious or chronic diseases.

## Introduction

**Figure 1:**
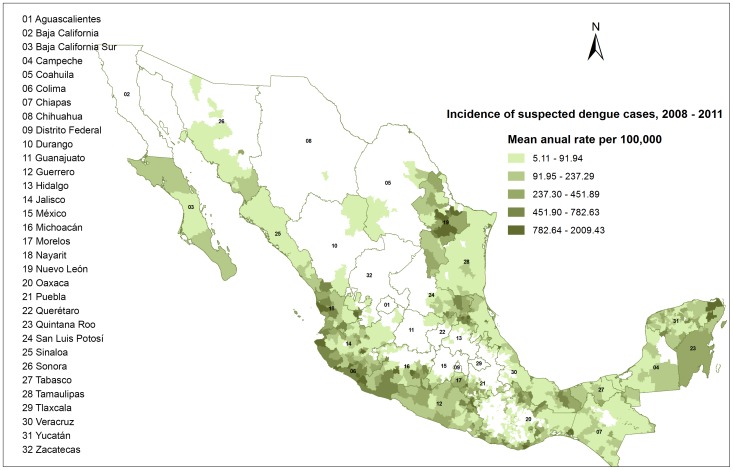
Incidence of dengue cases in Mexico during the period 2008 to 2011. Dengue cases have been reported in 30 of the 32 Mexican states. Geo-referenced case reporting started in 2008 when the EPS began interoperating with Dengue-GIS.

Dengue Fever (DF) is caused by the infection with four subtypes of Dengue virus and sometimes progresses to a severe and potentially deadly condition (Dengue Hemorrhagic Fever, DHF). Dengue viruses are transmitted to humans by the bite of infected mosquitoes (*Aedes aegypti* and *Ae. albopictus)*. The incidence and geographical dispersion of this disease expanded from nine countries in 1970 to more than 100 at present. More than 2.5 billion people are at risk with 50–100 million infections each year (1.6 million reported in the Americas in 2010) [1], with an estimated average cost of $2.5 billion US dollars per year [2]. Dengue fever is endemic in Mexico [3] where transmission is reported in 28 of its 32 states (Figure 1).

At present, the only public health strategy available for dengue control is directed against the mosquito vector populations. Effective control strategies require the intervention of multiple agencies within the health and other government sectors [4]. Access to timely and reliable epidemiologic and entomologic information is essential to support decision making and facilitate the interaction between the participating stakeholders. The lack of reliable and timely data useful to the different decision and operative components and levels of the control programs is the most important shortcoming in dengue control. For this purpose, national electronic reporting systems were recommended for surveillance, early detection and prediction of outbreaks [5].

Global dengue surveillance systems currently available include DengueNet, the World Health Organization’s central data management system, for the global epidemiologic and virologic surveillance [6]. Additionally, DengueMap [7], developed by CDC in collaboration with Health Map, uses Geographical Information System technology (GIS) to monitor DF transmission around the world. The gross detail and delayed data release of these systems limit their use by national control programs. GIS technology, based on the prevalence of suitable mosquito breeding sites and epidemiologic data has been used to assess the distribution and dynamics of mosquito populations or the risk of dengue and DHF in human populations [8]–[11], but to our knowledge, no national comprehensive electronic surveillance systems with the level of geographical disaggregation as the one presented in this work are currently available.

The Mexican public health system that provides dengue surveillance, preventive and control services is managed by the Federal Ministry of Health (MoH) in collaboration with the 32 decentralized State Health Services (SHS); these in term are organized in local health systems called sanitary jurisdictions. Additionally, Social Security Institutions (organized in delegations) provide services for a limited portion of the population working in the formal economy or state workers [12]. DF surveillance is coordinated, at the federal level, by the General Directorate for Epidemiologic Surveillance (GDES) and operated by the SHS through the State Epidemiology Departments[13]–[15]. A public health laboratory network diagnoses samples from patients with suspicion of dengue (probable DF cases) and a portion of these samples is analyzed for virus isolation (confirmed DF cases). Entomologic surveillance and vector control activities are coordinated by the National Center for Preventive Programs and Disease Control (NCPPDC) [13]–[15] and carried out by vector control departments in the SHS.

A web-based information system (Epidemiologic Surveillance Platform, ESP) was implemented in 2008 to collect data on suspected cases but it had no systematic means for data sharing and/or utilization; epidemiologic and entomologic surveillance and control information had no spatial disaggregation and flowed vertically to GDES and NCPPDC with little or no feedback to local health levels. This situation caused a proliferation of parallel local systems to support local decision making processes. An analysis of the available epidemiologic surveillance and vector control information systems, as well as data and results from a study to assess the performance of the Mexican Health Information System [16] identified that its main shortcoming were the fragmentation and diversity of non-compatible, electronic and paper-based information systems.

**Figure 2:**
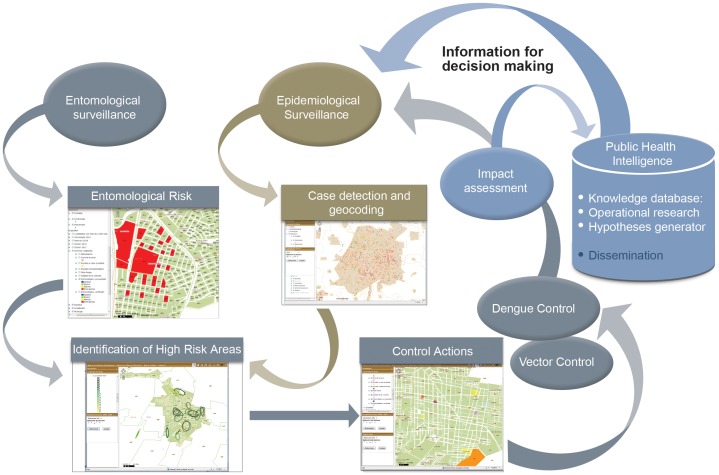
Conceptual Framework. Epidemiological and entomological data are entered and analyzed to produce risk maps which are then used to direct vector control activities. New epidemiological and entomological data are used to assess the impact of control activities thus generating a knowledge database which can be used to evaluate cost-effectiveness of control measures, accountability and operational research. Control interventions are directed according to the risk maps and entered into the system for impact evaluation in the reduction of cases and/or vector population.

The capacity of web-based GIS applications to facilitate timely registration of data, its integration and analysis could increase the capacity of national programs for dengue surveillance and greatly assist in its prevention and control [17] **.** We present herein a web-based GIS, with nationwide coverage for dengue surveillance and control based on the conceptual framework presented in Figure 2. It registers data collected at the local level and provides capacities to integrate and analyze information, such as spatial and time aggregation of DF cases, relevant to each level of the health system. The strategy to recruit the participation of health officers at different levels of the system for its design and construction could guide the implementation of similar systems in mid and low income countries.

## Methods

### Consensus

The format and contents of the new surveillance system were designed in consultation with experts in public health surveillance, disease control and prevention from the federal level and their respective counterparts in the SHS, as well as Information Technology (IT) developers. A detailed analysis of the available epidemiologic surveillance and vector control information systems and data and results from a study to assess the performance of the Mexican Health Information System [16] were used to identify shortcomings.

### GIS Basic Platform

Dengue-GIS was constructed as a web based information system with three tier architecture [18]. The Graphic User Interface (GUI) was developed in Microsoft ASP.Net 3.5 ^©^ based on MS-ISS 6.0 ^©^. The Model tier is based on ESRI ArcGis Server [19] and the data tier is based on MS-SQL Server ^©^. It is hosted at the National Institute of Public Health (NIPH) site on a server farm, totaling 32 cores for map production, eight cores for data base management and four for the internet server; each core with 4 GB ram. The entire site has an internet connection with a bandwidth of 30 Mb/s which is shared with NIPH’s institutional web applications.

To provide means for compatibility with the existing electronic information systems and future developments, a location ID-coding system was adopted. This is based on the National Geo-statistical Framework (NGF) from the National Institute for Statistics and Geography (NISG). The NGF is a unique geo-referenced identification system used to reference census data and other statistical data to geographic locations and geo-political features in the Mexican territory [20]. Additional geo-referencing capacities were obtained with the integration of a cartographic database developed by the Federal Electoral Institute (FEI), also based on the NGF, but with greater urban geographic detail. The combined cartography covers small rural towns with attributes, such as public and private schools (based on the Ministry of Education’s database) street centers and names and postal code among others. ID codes were assigned to town blocks, town block groups, census tracks, towns, cities, municipalities and states. A unique ID code was adopted to identify and geo-reference health units (Uniformed Code of Health Establishments, UCHE). This ID code was developed by the General Directorate of Health Information of the MoH. The final GIS contains the location of all public and private schools, the location of all health units managed by the public health institutions in Mexico (serving more than 90% of the population) as well as the total population and their socio-demographic characteristics, grouped by block, census tract, town, municipality state, region and nationwide. Other geographical data such as elevation, average temperature and precipitation is also available in the system at different scales.

### System Development

The GIS for surveillance and control of DF (Dengue-GIS) was constructed in three phases. In the first phase, an interoperable geocoding web based tool with a mapping application, designed to work with the EPS, was developed to geocode the place of residence (town blocks level) of probable dengue cases. Then, a modification was made on the EPS to enable geocoding through online calls in order to geo-reference the residence of probable cases. The EPS is now operated nationwide by the jurisdictional, state and social security system epidemiologists in coordination with the GDES.

The second phase included the development of a set of modules for entomological surveillance, planning and monitoring control activities, as well as a module for downloading information to the hard drive of local entomologists’ and epidemiologists’ computers. Vector surveillance is based on ovitrap data; for this purpose, a modification of the geocoding tool was developed to geocode ovitraps. After an ovitrap is geo-referenced, data on egg counts are entered into Dengue-GIS via web based formularies. Another set of web based formularies was developed to allow entering data on vector control activities carried out at the local level. Also, Dengue-GIS allows entering data gathered in entomological surveys, such as the number of water containers per house, the number of positive containers per house and the number of pupae-positive containers per house. These formularies are linked to town blocks using the NGF and the federal electoral identification codes. The modules for control and planning include tools to visualize temporal and geographical accumulation of cases (clusters of DF transmission) in order to direct vector control activities. It also includes a tool to print maps to be used in field activities; these maps have the id code of each block to allow recording of surveillance/control activities.

The third phase was completed with a reporting module and an improved geographic visualization tool that combines epidemiologic, entomological and control data into a single mapping window.

Each of these phases was implemented gradually in the states with most DF incidence, and a group of researchers and IT specialists, along with GDE personnel trained state and jurisdictional epidemiologist in its use.

### Statistical Analyses

Identification of areas of probable cases accumulation in time (clusters) is carried out using a nearest neighbor hierarchical clustering algorithm [21]. This statistical methodology is used in epidemiology to detect areas of high accumulation of cases based on the distance between them. For its application in Dengue-GIS, a minimum number of cases to conform a cluster was set to five and the maximum distance between any two cases to 500 meters. The algorithm is run every week using the EPS geo-referenced epidemiological data of the three previous weeks. In this way the entire national territory is scanned every week in search for areas with an accumulation of at least five cases separated by 500 meters or less within a three weeks’ time window. The resulting graphical output of the algorithm is an ellipse, centered at the geographical centroid of the set of locations (points) where the probable cases were geocoded. The length of axes of the ellipses is determined by the standard deviation in the north-south and east-west dispersion of the location of the cases. The orientation of the ellipse is based on the directional mean of the set of locations to where the cases were geocoded. The directional mean is determined by an angle that minimizes de distance of every point in the cluster to both axes of the corresponding ellipse. Every week cumulative incidence rates are calculated at a micro-regional scale to help in the detection of high risk areas. Historical information on the yearly incidence per micro-region is also available (shown in File S1 Annex 1). Significance of each week clustering scheme is tested using Monte-Carlo simulation with 100 repetitions.

A risk of transmission index was developed in collaboration with the national vector control personnel, combining the ovitrap contents data and the epidemiological surveillance data. According to the entomological surveillance norm, four ovitraps are to be placed in each city block under surveillance. City blocks are classified into four entomological risk categories, according to the mean number of eggs per ovitrap using the city specific quartiles of the mean counts distribution as cutoff points: low, moderate low, moderate high and high. A 300 meter radius buffer (circle), centered at the geographical centroid of the location of the four ovitraps of each block, is drawn and geographically intersected with the epidemiological surveillance data (points representing the location of cases) to produce the transmission risk index:

- High transmission risk = high entomological risk and presence of dengue probable cases
- Moderate high transmission = Moderate high entomological risk and presence of dengue probable cases
- Moderate low transmission = Moderate low entomological risk and presence of dengue probable cases
- Low transmission risk = no dengue probable cases detected

## Results

### Epidemiological Surveillance

**Figure 3:**
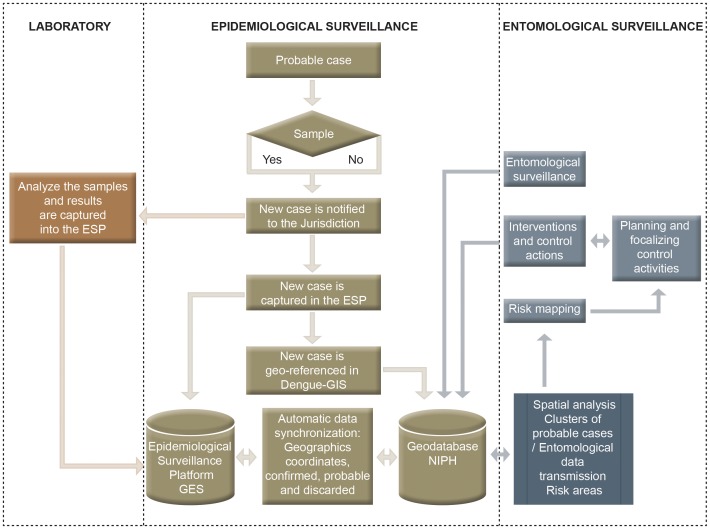
Web-based geographic Information System for integrated dengue epidemiologic surveillance and control data flow diagram. Epidemiological data on probable case is entered into the system at the health center or sent in paper form to the sanitary jurisdiction where is entered into the EPS system. The case is simultaneously geo-coded and data stored in the geodatabase. Laboratory results from blood samples, taken to a portion of the probable cases, enters the system and serves to update the EPS and Dengue GIS data base. Entomological surveillance data and control activities are entered into the Geodatabase by vector control personnel. The integration of these data is used to produce risk maps.

According to Mexican official norms, epidemiologic data on dengue patients is entered into the surveillance system within one week after the patient’s first contact with the health system and it is reported by epidemiological week. New cases detected by primary care providers are notified (using a probable-case-registration form) to the lowest administration level (health jurisdiction) where data is introduced in the EPS/Dengue-GIS. The geocoding system is very intuitive and, when invoked opens a new window displaying a map of the area of residence (based on the address and the NGF) of the probable case. Clicking on a button invokes an instance of the geocoding tool that displays in the computer screen a map of the area based on state, municipality, city, and postal code. To simultaneously register in the EPS and geocode probable dengue cases, the local epidemiologist clicks with the cursor in a town block, using the address information on the probable-case-registry (Figure 3). The probable case information (without any personal data) is then sent to a geo-database for processing and mapping. The screen coordinates are transformed into geographic coordinates and stored at a geocoded data base (at NIPH) and the EPS data base (at GDES) servers. The epidemiologist or health provider has to click only on the screen to record the coordinates and the map window closes to return to the EPS screen. With this simple process the data can be analyzed in a geographical domain with very high resolution. Geo-coding is done at the block level to avoid disclosing the actual place of residence of probable dengue cases.

**Figure 4:**
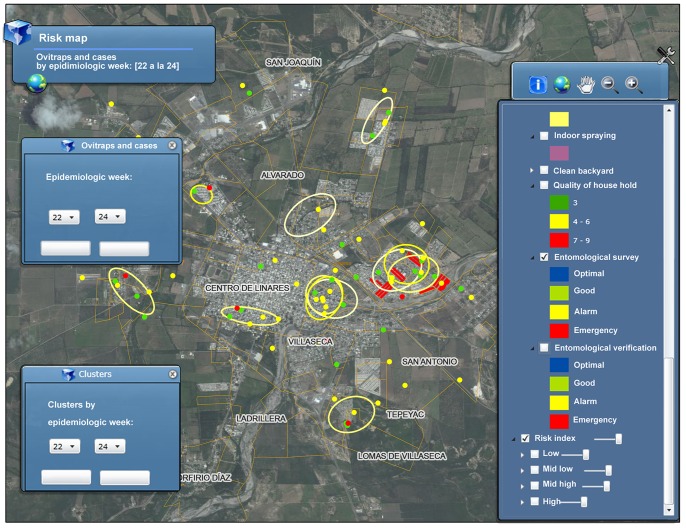
Screenshot of Dengue-GIS application. Dengue-GIS screenshot showing the distribution of probable cases and case clusters (high transmission areas) during the CDC weeks 22–24 2012 in Linares City, Nuevo Leon. Locations of entomological survey activities during the same period are shown in red. DF cases accumulation in space and time is showed by a set of ellipses which represent the graphical output of the cluster detection algorithm. Two areas, one very close to downtown Linares, and the other to the west of the city show three intersecting ellipses, this indicates that transmission occurred uninterrupted during the three week period showed. Cases are represented by dots; red dots are laboratory confirmed cases, green dots are cases discarded by laboratory tests (other disease) and yellow dots are those for which there is not available laboratory data (either because it was not selected for blood test, according to surveillance protocol or because the public health laboratory has not yet got/entered the respective results). Red squares represent the city block where the entomological surveys were carried out, showing emergency results. Entomological surveys are a separate surveillance activity in which information about the conditions of households is collected. Pictures presented here were modified to translate the text presented to English; the actual Dengue-GIS is an all-Spanish language system.

As part of the regular epidemiological surveillance protocol and according to the Mexican official norms [13], [14], blood samples for diagnosis are drawn to a variable portion of dengue probable cases. This blood samples are obtained at the point of care by the health care service providers and sent to the public health laboratory. Data on laboratory case confirmation is entered by the laboratory personnel into the EPS and case status is automatically updated in Dengue-GIS through the internet connection after each registration (Figure 3). Figure 4 shows the location of cases, as color coded dots, in the city of Linares, Nuevo León during the epidemiologic week 22 to 24 of the year 2012. Red dots represent confirmed cases, green dots represent discarded cases (not dengue cases) and yellow dots represent probable cases for which no lab info is available (either because the public health lab has not yet entered their results or because it was not selected for a blood sample according to the surveillance protocol). Every Wednesday, an automated process to detect areas of probable dengue cases concentrations is conducted using the epidemiologic surveillance data of the three previous weeks. Figure 4 shows the graphical results of this process for the weeks 22 to 24 of 2012 as yellow ellipses. In the center of the figure, two areas in which three ellipses intersect indicates that the accumulation of cases occurred, consecutively, during the three weeks showed. For the purpose of display, ellipses are also color coded by epidemiological week in which they appeared: green at the beginning of the year degrading to yellow by mid-year and to purple by the end of the year. This allows the visual analysis of longer periods and the identification of areas where transmission is persistent. File S1 is an animated graphic representation of a longitudinal follow up depicting Merida city blocks with DF cases, the distribution in time and space of clusters of case accumulation in relation to the overall case incidence in 2012, as well as vector control interventions.

### Entomological Surveillance and Vector Control

Entomological surveillance is based mainly on mosquito ovitraps, but the system can be used for the inclusion of other parameters, such as numbers and locations of mosquito breeding sites based on the household and/or environmental conditions [13]–[15], [22], The local dengue program operative personnel perform weekly monitoring of mosquito ovitraps (positivity and number of eggs per trap) and enters into Dengue-GIS the individual results at the jurisdiction administrative offices (Figure 3). Entomological data are geocoded to the respective town block and stored in the Geodatabase (NIPH). Anti-vector interventions are registered in the same manner. Data on entomological surveillance and vector control activities are systematized and displayed in working maps readily available to the local and federal control levels. Figure 4 shows the results of entomological surveys from the east of downtown Linares (center of the figure); the color coded blocks, red and yellow, indicate that those areas are in high risk (alarm and emergency). These areas are precisely where dengue cases clustered for the three consecutive weeks showed in the figure.

**Figure 5:**
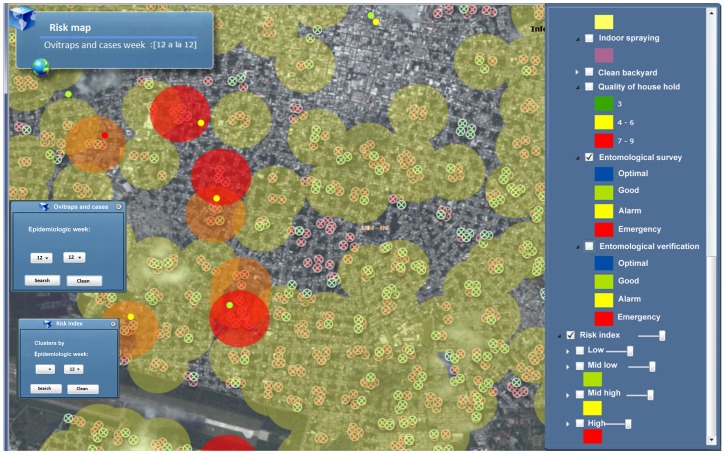
Screenshot of Dengue-GIS application showing the transmission risk index. Every Tuesday an evaluation of the abundance of the vector population, estimated by the mean number of eggs per city block and the presence of probable dengue cases is carried-out. The color coded circles represent areas of differential dengue transmission risk: high (red), moderate-high (orange) and moderate-low (yellow). Low risk areas are not shown.

Every Tuesday entomological data maps are constructed and used for the estimation of transmission risk. Figure 5 shows the graphic output of this process for the city of Merida, Yucatan for the epidemiological week 12 of 2013. The location of ovitraps with *Ae. aegypti* eggs are represented by red dots. The various degrees of risk are represented by yellow, orange and red circles. For simplicity, low risk areas are not shown in the map.

### Dengue-GIS Use

The Information system for epidemiological surveillance (EPS) interoperates on real time with Dengue-GIS (Figure 3). The integration of geo-referenced data on dengue cases, entomological parameters and control activities makes it possible to evaluate identify and prioritize risk areas (Figures 4 and 5).

Dengue-GIS is available for the federal dengue control program leaders, state program leaders and the operative personnel in all (236 nationwide) sanitary jurisdictions. It is also available to all personnel in charge of epidemiological surveillance at all levels of the health system. Almost two years after starting its implementation Dengue-GIS has more than 302 active users in 31 states, with an average of 204 users during a working day. A total of 147,495 ovitraps have been deployed, geo-referenced and weekly monitored in 526 large cities (where more than 36 million people live) in the states with historically high dengue incidence; covering important cities such as Guadalajara in Jalisco, Monterrey in Nuevo León, Cuernavaca in Morelos., Merida in Yucatán, Cancun in Quintana Roo and Acapulco in Guerrero.

The system has accumulated a total of 6′ 667,385 records. Up to the 48^th^ epidemiological week in 2012 there were 297,797 geo-referenced probable cases in the system (42% of all cases registered in the EPS). The proportion of geo-referenced cases are discretionally decided by control programs on the bases of numbers of suspected cases within geographic locations and the surveillance intensity necessary for the identification of outbreaks, assessment of transmission levels, and control monitoring. With this epidemiological information, 40,687 clusters of probable transmission have been identified scattered in the national territory, since 2010.

According to the laboratory results on patient blood samples (33% of all cases registered in the EPS), the positivity index is 33.1% (26.1% –38.1%) in the period 2008–2012, thus an estimate of 237,748 confirmed cases registered.

There are 449,295 records with control activities by town block in 8,120 urban and rural areas where more than 54 million people live 48.5% of the country’s population, (39.08% in urban areas and 9.45% in rural areas). Currently, this information system registers approximately 84,798 records every week including probable cases and lab confirmation data, ovitrap readings, control interventions (insecticide nebulization, indoor spraying, and larvicide application) as well as the transmission clusters (Figure 4).

## Discussion

The main features of Dengue-GIS is its seamless capability to integrate, in an interoperable web based platform, the basic information needed for problem appraisal and control planning, which is readily available to users at all levels of the control program scaffold. Dengue-GIS includes tools for the integrated analyses of epidemiological and entomological surveillance for the identification of increased transmission areas and to monitor vector control activities. The availability of an operable (timely and spatially disaggregated) epidemiological stratification, along with the capability to assess the effect of focalized control interventions, provide local and federal control programs with information for evidence-based decisions within a public health intelligence platform (Figure 2).

Previous to the EPS and Dengue-GIS implementation, epidemiological data, produced locally by health providers, travelled each week in paper forms to their respective sanitary jurisdictions and then to the State Epidemiology Department. The data was then entered into a local electronic system, which in term produced files to be sent to GDE either by disk in the early 90′s or more recently by e-mail. GDE compiled all the data into a single database and produced weekly epidemiological reports. Epidemiological and entomological surveillances were unconnected and the information flow was slow. As in most endemic countries, dengue surveillance was of little use in spite of the amount of work required for data collection and registration; the data flow and the aggregation process limited the capacity for local analysis impeding its use for anti-dengue operations. Nonetheless, epidemiological data was collected and at best used for planning next season control operations; while entomological surveillance was mostly used to assess *a posteriori* anti-mosquito interventions.

With Dengue-GIS is now possible to identify at nation-wide scale where and when accumulation of probable dengue cases are occurring (Figure 4) but with a spatial resolution suitable to direct and evaluate vector control activities. The use of cluster detection analyses and risk assessment on a weekly basis produce timely information for decision making at all levels of the dengue control program. For example, the visualization of areas in which accumulation of cases occurred in three consecutive weeks (Figure 4), makes it obvious that some control actions are there needed immediately.

Putting apart the construction of the web site, developing Dengue-GIS implied a complex process. It began with the incorporation in its design of the required components already in use and new ones of possible use by the vector control programs. Operatively, the construction of data input screens required to be as simple as possible and of friendly interaction. Also, the adoption of the system for the routinely registration of data and utilization of integrated surveillance reports required changes in the perception about their usefulness and in the operative culture of its potential users.

The case detection efforts, according to the prevailing local epidemiology, remained the same with the introduction of Dengue-GIS. Basic information related to dengue cases (age, sex, date, address) conserved the traditional usage, but data registration and consultation was facilitated by user-friendly screens. Although weekly epidemiological reports are still produced (in accordance to the specific action program 2007–2012) [22], it has become web based. This reports are now available at http://www.epidemiologia.salud.gob.mx/dgae/panodengue/intd_dengue.html. EPS and Dengue-GIS implementation also introduced other improvements for epidemiological surveillance as the simultaneous registration and availability of the data from local to federal databases and thus the capability of close monitoring in almost real time at all administrative levels of the health system. With several geocoded data layers including environmental, demographic, health system infrastructure and accessibility, Dengue-GIS provides a wider panorama for the identification of the determinants of the disease transmission. Additionally, historical epidemiological data can be easily consulted at different levels of spatial and time aggregations to monitor changes in the intensity of transmission. Laboratory results are now readily available and are mapped in real time. This is a very important improvement for epidemiological surveillance and for the planning and evaluation of vector control activities.

The geo-codification of entomological data required the introduction of new processes in the surveillance activities of the control programs. Data input screens facilitated de registration of the main entomological indicators based on breeding site characterization, and the adoption of a surveillance strategy based on a network of geo-coded ovitraps required new data enter formularies. In addition, the capacity of Dengue-GIS to register anti-vectorial interventions, that can be displayed and analyzed along with the mosquito monitoring information, serves as an input for the analyses of impact and cost-effectiveness of their interventions both in the human and vector populations. Similar to case registration, entomological and control data is simultaneously available to all levels of the control program. The system’s capacity to perform spatial-temporal analyses integrating entomological and epidemiological data provides evidence (easily consulted in screenshots) for planning and conducting vector control activities based on risk assessment (Figures 4, 5).

Currently, the national vector control program has adopted the risk based strategy to carry out vector surveillance and control activities, including in its operative guidelines for entomological surveillance the use of the information generated by Dengue-GIS (http://kin.insp.mx/aplicaciones/Plataformadengue/Nacional/Capacitacion/Estrategias%20Dengue/Estrategia_Control_focalizado.pdf accessed 17 april 2013).

The several processes involved in Dengue-GIS development and its implementation present common situations in other dengue endemic countries where traditional surveillance requires modernization to upgrade its usefulness in control and prevention (5). The organizational inertia, built over decades, is probably the most important source of the obstacles faced during the introduction of new procedures [23], [24]. To build on ownership, a trans-disciplinary team with experts in dengue surveillance and control from the federal and state levels, as well as local public health workforce. Consensus among all stakeholders on the need to use and share geo-referenced data to facilitate prediction and early outbreaks detection, as well as to evaluate the impact of control activities was achieved. The plasticity of the system allowed the inclusion of new components for compliance to requests made by local epidemiologists and entomologists.

Resistance to change is related to the amount of modifications introduced into current practices [24]. Implementation of Dengue-GIS was facilitated by the adoption of already working information systems as its building blocks. Although the changes introduced in the EPS were minimal, the fragmented health system and the diversity of data management protocols required a great deal of promotion to convince health and vector control personnel about the benefits of geo-coded data surveillance.

The use of Dengue-GIS is now at full scale in Mexico and the data produced by epidemiological and entomological surveillance is used to direct vector control actions. In a self-applied questionnaire answered by the national program leader, nine state leaders and 158 users (operative personnel at the jurisdictional level) of 27 states, 78% found Dengue-GIS amiable, 82% indicated that it provides useful information for the conduction of their activities and 84% uses the transmission clusters to focalize control actions and to visualize coverage of prevention and control activities. Also, 86% considered that Dengue-GIS has been beneficial to the overall management of their activities. State leaders reported that having online access to information helps them to keep track of prevention/control activities and that the system is helping them to strengthen dengue control.

The continuous use of Dengue-GIS for data collection and analyses will eventually generate a population based knowledge database for the development of operative research projects and could be used to identify cost-effective interventions in specific operative settings. This geo-referenced population database can also be useful, in phase III trials of the incoming dengue vaccines [25]–[28].

Some unexpected uses of the system have begun to appear; the states programs are using Dengue-GIS in different applications for new types of analyses; for example, Jalisco aggregates data to produce risk maps by different geo-political units; Morelos generates maps of insecticide nebulization coverage (as recorded by GPS installed in the fogging machine) which in term could be incorporated into Dengue-GIS. The accessibility to an accumulated set of epidemiological and control assessment data has motivated the scientific community to document and get data for research projects and currently four projects are using data and the spatial capabilities of the system. Its capacity to record health interventions opens the possibility to extend the use of its platform to the surveillance and monitoring and evaluation of other health programs activities.

Integrated Dengue Surveillance systems such as the one presented here are now feasible; they must be part of the prevention and control of this disease. Digital cartography and automated address locators have different development levels in countries where dengue is endemic. Poor urban planning and unregulated urban expansions, along with the lack of national standards to record addresses make it very difficult to automate epidemiological and entomological data geocoding. Nonetheless, these limitations can be solved with tools like Google maps and Google earth and their Application Programing Interfaces (API) [29]. Similarly, open source GIS software such as GRASS and MapServer, a desktop application and web map server respectively [30], [31], as well as a growing set of spatial analytical tools, like the *spatial view* in the statistical programming language R [32] could be integrated in these surveillance systems.

## Supporting Information

File S1 Flash movie showing the longitudinal nature of the epidemiological, entomological and control data collected through DENGUE-GIS. In this interactive movie the probable case clusters, as well as the control actions are shown by epidemiological week on a micro-spatial scale in the city of Merida Yucatan. The epidemic curve is also shown for reference. The user can play the movie for the 52 epidemiologic weeks and also pause it or play it backwards to better understand the relation of the control actions, the transmission clusters and the epidemic curve.
(SWF)
