# Correction: Nation-Wide, Web-Based, Geographic Information System for the Integrated Surveillance and Control of Dengue Fever in Mexico

**DOI:** 10.1371/annotation/fa2d8273-9377-44c0-b61f-eb019beca2ce

**Published:** 2014-01-16

**Authors:** Juan Eugenio Hernández-Ávila, Mario-Henry Rodríguez, René Santos-Luna, Veronica Sánchez-Castañeda, Susana Román-Pérez, Víctor Hugo Ríos-Salgado, Jesús Alberto Salas-Sarmiento

Juan Ignacio Arredondo-Jimenez was not included in the author byline. He should be listed as the eighth author and affiliated with National Center for Preventive Programs and Disease Control, Mexico, D.F. 

The contributions of this author are as follows: Contributed reagents/materials/analysis tools, Other: designed the method of calculating spatial risk areas.

